# Case Report and Review of the Literature: Congenital Diaphragmatic Hernia and Craniosynostosis, a Coincidence or Common Cause?

**DOI:** 10.3389/fped.2021.772800

**Published:** 2021-11-26

**Authors:** Linda Gaillard, Anne Goverde, Quincy C. C. van den Bosch, Fernanda S. Jehee, Erwin Brosens, Danielle Veenma, Frank Magielsen, Annelies de Klein, Irene M. J. Mathijssen, Marieke F. van Dooren

**Affiliations:** ^1^Department of Plastic, Reconstructive and Hand Surgery, Erasmus Medical Center—Sophia Children's Hospital, University Medical Center Rotterdam, Rotterdam, Netherlands; ^2^Department of Clinical Genetics, Erasmus Medical Center—Sophia Children's Hospital, University Medical Center Rotterdam, Rotterdam, Netherlands

**Keywords:** case report, craniosynostosis, congenital diaphragmatic hernia (CDH), BCL11B, craniosynostosis syndromes

## Abstract

Congenital diaphragmatic hernia (CDH) is a life-threatening birth defect that presents as either an isolated diaphragm defect or as part of a complex disorder with a wide array of anomalies (complex CDH). Some patients with complex CDH display distinct craniofacial anomalies such as craniofrontonasal dysplasia or craniosynostosis, defined by the premature closure of cranial sutures. Using clinical whole exome sequencing (WES), we found a *BCL11B* missense variant in a patient with a left-sided congenital diaphragmatic hernia as well as sagittal suture craniosynostosis. We applied targeted sequencing of *BCL11B* in patients with craniosynostosis or with a combination of craniosynostosis and CDH. This resulted in three additional *BCL11B* missense mutations in patients with craniosynostosis. The phenotype of the patient with both CDH as well as craniosynostosis was similar to the phenotype of previously reported patients with *BCL11B* missense mutations. Although these findings imply that both craniosynostosis as well as CDH may be associated with *BCL11B* mutations, further studies are required to establish whether *BCL11B* variants are causative mutations for both conditions or if our finding was coincidental.

## Introduction

The genetic etiology of congenital diaphragmatic hernia (CDH) is complex. Structural variants, small and large insertions or deletions and single-nucleotide variants (SNVs) in over a 100 genes have been associated with CDH (1). Only a few of these genes are mutated recurrently in multiple individuals and even then the phenotype can differ largely due to incomplete penetrance. CDH has a prevalence of 2.3–2.7 per 10.000 live births (2–4) and can present as isolated CDH or in association with additional congenital anomalies (non-isolated CDH or complex CDH), as seen in ~40 (5) to 49% (6) of the cases. Complex CDH may present as part of a recognizable genetic syndrome, chromosome abnormality, or a collection of major congenital malformations. One of the less common malformations in patients with CDH are craniofacial anomalies and in particular craniosynostosis.

Although less common in syndromes associated with complex CDH, several studies have described syndromes such as Apert's syndrome and Craniofrontonasal syndrome, that include both CDH as well as craniosynostosis as cardinal or relatively common features (7–22). Craniosynostosis, a developmental disorder defined by the premature fusion of one or more sutures, has a prevalence of 7.2 per 10,000 live births (23). It can be divided into non-syndromic and syndromic craniosynostosis, with syndromic craniosynostosis being characterized by additional congenital anomalies, such as limb anomalies and neurodevelopmental delays (24, 25). As part of our standard clinical care, all patients diagnosed with craniosynostosis are offered targeted genetic analysis (26). In a female patient with both CDH as well as craniosynostosis, we observed a genetic variant of the B cell leukemia 11b gene (*BCL11B;* OMIM 606558) resulting in an amino acid change at position 667 [p.(Gly667Glu)].

Searching the literature for BCL11B mutations we found several case reports. First, a case report described a male patient with a heterozygous *de novo* missense *BCL11B* mutation (p.Asn441Lys), who presented with severe combined immunodeficiency as well as neurological, dermal and facial dysmorphisms including hypertelorism, short palpebral fissures and micrognathia (27). Second, a study reported on 13 patients with heterozygous germline mutations in *BCL11B* (28). Most of these patients presented with neurodevelopmental disorders and immunodeficiency with reduced type 2 innate lymphoid cell and were carriers of loss of function mutations in BCL11B. However, in the most severely affected patient (patient EII-I) a missense BCL11B mutation p.(Asn807Lys) was found. This patient had a similar phenotype as compared to the first patient described by Punwani et al. (27) including a myopathic facial appearance, hypertelorism and small palpebral fissures. Neither study reported on the presence of craniosynostosis or CDH although craniofacial anomalies were described for patient EII-I and are apparent for the patient described by Punwani et al. (27). However, we previously discovered a *de novo BCL11B* missense mutation in exon 1 which encodes for [p.(Arg3Ser)] in a male patient with unilateral coronal suture craniosynostosis (29). In addition, a *de novo BCL11B* missense mutation in exon 4 [p.(Arg350Cys)] was reported in a patient who presented with CDH, an abnormal optic nerve, increased intraocular pressure and scoliosis (30). These findings suggest that both craniosynostosis as well as CDH may be associated with *BCL11B missense* mutations.

For this report, we selected patients who had undergone genetic testing with craniosynostosis or combined CDH and craniosynostosis from the Erasmus MC- Sophia Children's hospital. This search resulted in three additional *BCL11B* missense variants. In this report we present the clinical reports of these four new patients with *BCL11B* variants and a brief review of disorders that are characterized by both CDH as well as craniosynostosis. Informed consent to publish case descriptions and images was obtained from patients, and/or their parents if applicable. All genetic information is provided according to the HGNC guidelines (31).

## Clinical Reports

### Patient A

**Figure 1:**
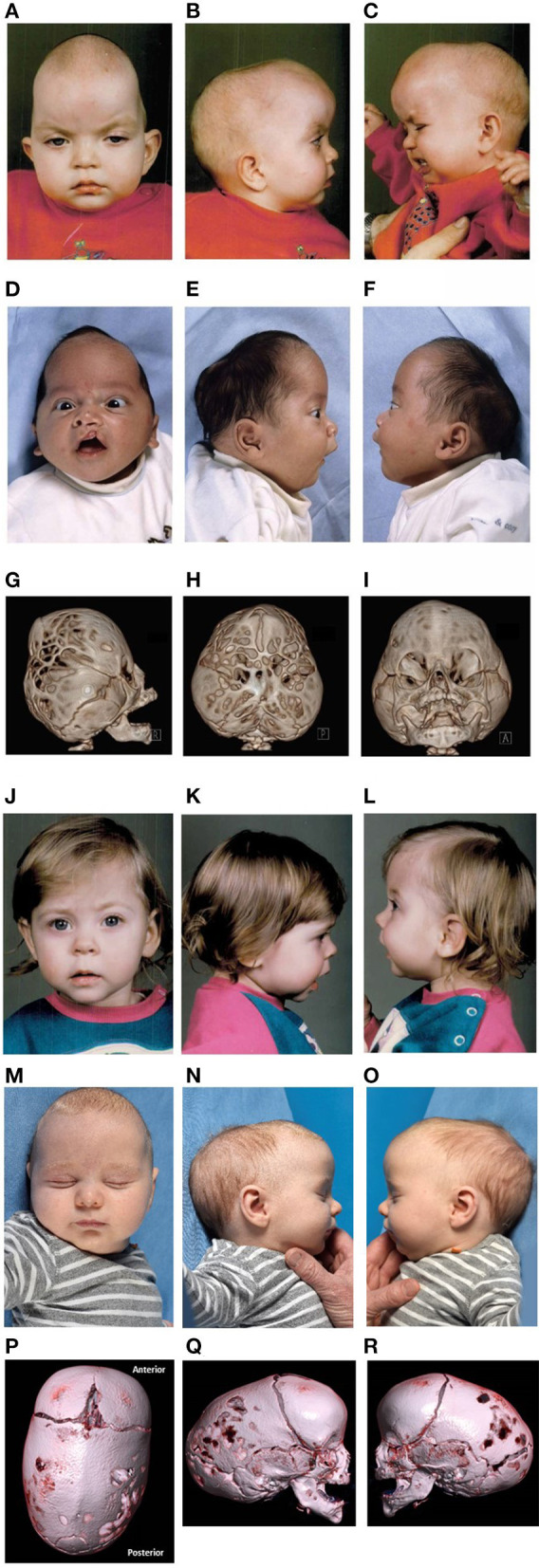
Clinical features. Photos published with consent. **(A–C)** Pre-operative features of patient A at the age of 14 months. Historic pre-operative radiological imaging could not be retrieved of the CDH and craniosynostosis. **(D–I)** Pre-operative features and 3DCT-scan imaging of patient B at the age of 1 and 6 months, respectively. **(J–L)** Pre-operative features of patient C at the age of 12 months. Historic pre-operative radiological imaging of the craniosynostosis could not be retrieved. **(M–R)** Pre-operative features and 3DCT-scan imaging of patient D at the age of 2 months.

Patient A [NM_138576.3(BCL11B):c.2000G>A, p.(Gly667Glu)], a 30 year old female, is the third child of healthy non-consanguineous parents, born at 41 weeks of gestation with a birth weight of ~2,200 g, following an uncomplicated pregnancy. Shortly after birth, the patient developed severe respiratory insufficiency, resulting in apnea and asystole. After resuscitation, the patient was transferred to our tertiary care center. She presented with left-sided CDH, resulting in respiratory insufficiency and pulmonary hypertension, for which she underwent surgery the second day after birth. She was weaned off respiratory support after 2 weeks. She showed several dysmorphic features including, slight ocular proptosis, hypertelorism, down slanting palpebral fissures, ptosis, arched eyebrows, and syndactyly of the second and third toes of both feet (Figures 1A–C). In addition, she suffered from feeding difficulties and gastroesophageal reflux due to pyloric stenosis, which was surgically corrected at the age of 2 months. She remained hypotonic and developed psychomotor delays during the first year of life. At the age of 11 months she was diagnosed with progressive sagittal suture craniosynostosis, for which she underwent surgery at the ages of 14 and 16 months. During surgery part of the calvarium looked abnormal and was sent for pathological investigation, revealing a capillary and cavernous hemangioma. She developed divergent strabismus, latent nystagmus, hypermetropia and astigmatism, and suffered from recurrent episodes of pneumonia, sinusitis and rhinitis. She showed no signs of severe cognitive impairment.

### Patient B

Patient B [NM_138576.3(BCL11B): c.1744G>A, p.(Gly582Ser)], a 16 year old male, is the second child of unaffected non-consanguineous parents. He was born at 40 weeks of gestation, with a birth weight of 3,710 g. He presented with craniosynostosis of the sagittal suture and both lambdoid sutures, for which he underwent surgery at the age of 6 months. In addition, he suffered from an incomplete left-sided unilateral cleft lip including the alveolar arch, which was surgically corrected in three stages (at the ages of 6 months, 6, and 11 years). He showed several dysmorphic features, including frontal bossing, down-slanting palpebral fissures, hypotelorism, mild webbing of the neck, and hyperpigmentation on the left shoulder (Figures 1D–I). Magnetic Resonance Imaging (MRI) of the brain at the age of 8 years showed a Chiari I malformation. Ophthalmological assessment revealed astigmatism and myopia. He developed a proportionate short stature of −2.5 to 2 SD with delayed bone age, for which puberty was postponed using leuprorelin and letrozole. He had no neurodevelopmental delays. Motor and speech development were within normal range although he required speech therapy and physical therapy. He developed Kawasaki disease at the age of 7 years, which responded well to intravenous immunoglobulins. At the age of 16 years he underwent a second cranial vault surgery including biparietal expansion and occipital decompression. He did not suffer from frequent infections.

### Patient C

Patient C [NM_138576.3(BCL11B):c.2018C>G, p.(Pro673Arg)], a 30 year old female, is the child of non-consanguineous phenotypically normal parents. A sibling and paternal half-sibling are both healthy. She presented with left-sided unicoronal craniosynostosis, for which she underwent surgery at the age of 14 months. She developed no neurodevelopmental delays and did not suffer from recurrent infections to our knowledge, although the cranial vault operation was postponed twice due to upper airway infections. She required glasses from the age of 8 years onwards. She did not display any evident dysmorphic features with the exception of mild vertical orbital dystopia (Figures 1J–L).

### Patient D

Patient D [NM_138576.3(BCL11B):c.1265C>T, p.(Pro422Leu)], a 2 year old male, is the second child of unaffected non-consanguineous parents. In addition, the patient has two unaffected paternal half-siblings. He was born with a birth weight of 4,230 g at 41.3 weeks of gestation. He was born with the umbilical cord wrapped around his neck but recovered well after stimulation and did not require oxygen support. The pregnancy was otherwise uncomplicated. He presented with craniosynostosis of the sagittal suture at the outpatient clinic at the age of 3 months for which he underwent spring-assisted surgery at the age of 6 months. He had no neurodevelopmental delays and did not suffer from recurrent infections. Dysmorphic features were mild and included thick alae nasi and mild retrognathia with an overbite (Figures 1M–R).

## Overview of Patients With BCL11B Missense Mutations and Variants

An overview of all patients described above, including genetic and phenotypic information of each patient can be found in Table 1. In addition, it shows the previously described patients with *BCL11B missense* mutations. Phenotypical features vary heavily among patients. The patient, reported by Goos et al. (29), displayed a similar phenotype to our Patient C, with both patients presenting with coronal suture synostosis without neurodevelopmental delays or other severe associated features. Patient A and patient B, are more similar to patients reported by Punwani et al. (27) and Lessel et al. (28). They have a more severe phenotype, presenting with a combination of multi-sutural craniosynostosis, CDH and cleft. In an additional study, one patient with a *de novo BCL11B* missense mutation [p.(Arg350Cys)] was reported to have presented with complex CDH (30). All cases summarized above have missense variants in exon 4 with the exception of the patient reported by Goos et al. (29), who had a *de novo* missense mutation of exon 1.

**Table 1:** Case descriptions of patients with BCL11B missense variants: genetic and phenotypical features.

	**Patient A**	**Patient B**	**Patient C**	**Patient D**	**Goos et al. (29)**	**Lessel et al. (28) (E:II-1)**	**Punwani et al. (27)**	**Longoni et al. (30) (T45)**
Nucleotide change*	c.2000G>A	c.1744G>A	c.2018C>G	c.1265C>T	c.7C>A	c.2421C>G	c.1323T>G	c.C1048T
Protein change	p.(Gly667Glu)	p.(Gly582Ser)	p.(Pro673Arg)	p.(Pro422Leu)	p.(Arg3Ser)	p.(Asn807Lys)	p.(Asn441Lys)	p.(Arg350Cys)
Exon	4	4	4	4	1	4	4	4
CADD score^‡^	18.89	3.944	22.7	22.6	24.5	25.5	24.8	31
Type of variant	Missense**	Missense**	Missense	Missense	Missense	Missense	Missense	Missense
Mode of inheritance	Maternal	Paternal	Maternal	Maternal	*De novo*	*De novo*	*De novo*	*De novo*
**Phenotype**								
**Gastro-Intestinal**								
CDH	Left-sided	–	–	–	–	–	–	+, complex
Feeding difficulties	+	–	–	–	–	+	NR	NR
Pyloric stenosis	+	–	–	–	–	+	NR	NR
Gastroesophageal reflux	+	–	–	–	–	+	NR	NR
**Skeletal**								
Craniosynostosis	Sagittal	Sagittal, lambdoid (bilateral)	Coronal, left	Sagittal	Coronal, right	–	–	NR
**Cognition, behavior, and motor development**								
Intellectual disability	–	–	–	–	–	+	+	NR
Speech impairment	+	Speech therapy required	–	–	–	+	+	NR
Delay in motor development	+	Physical therapy required	–	–	–	+	+	NR
**Dysmorphic features**								
Myopathic facial appearance	+	–	–	–	–	+	NR	NR
Eyebrow anomalies	Arched	–	–	–	Narrow	–	NR	NR
Small palpebral fissures	–	–	–	–	–	+	+	NR
Hypertelorism	+	–	–	–	–	+	+	NR
Hypotelorism	–	+	–	–	–	–	–	NR
Prominent nose	–	Asymmetric nose	–	Thick alae nasi	Short nose	+, upturned	NR	NR
Long philtrum	+	–	+	–	+	–	NR	NR
Lip anomalies	Full lower lip	Incomplete unilateral cleft lip and alveolar arch	Full lower lip	–	–	Thin upper lip and vermilion; down-turned corners, small mouth	NR	NR
Ptosis	+	+	–	–	–	NR	NR	NR
Downslant of the eyes	+	+	–, upslant	–	–	–, upslant	NR	NR
Eversion of the lower eyelids	+	+	–	–	–	NR	NR	NR
Vertical orbital dystopia	–	–	+	–	+	NR	NR	NR
Retrognathia	–	+, mild	+, mild	+, mild	–	Micrognathia	Micrognathia	NR
**Dysmorphic features**								
Dermatological anomalies	Eczematous skin	Hirsutism	–	–	–	Severe congenital erosive dermatitis	Erythematous psoriaform dermatitis hirsutism	NR
Ear anomalies	Low-set ears	Low-set ears, fleshy upturned earlobes	–	–	–	Posteriorly rotated ears	Ear tag	NR
Other	Deep-set eyes	Deep-set eyes	Mild asymmetry of the eyes; deep-set eyes; periorbital fullness	Deep-set eyes	-	Bitemporal hollowing, hypoplastic midface	Abnormal nasal creases, loose skin folds	NR
**Extremities**								
Anomalies of the hands	Brachydactyly (bilateral)	Short fifth digits (bilateral)	–	–	–	Non-congenital syndactyly	NR	NR
Anomalies of the feet	Syndactyly of the second and third toes (bilateral)	Syndactyly of the second and third toes (bilateral)	–	–	–	Non-congenital syndactyly	NR	NR
**Neurological**								
Hypotonia	+	–	–	–	–	+	+	NR
Unstable gait	+	–	–	–	–	+	NR	NR
**Ophthalmological**								
Refractive error	Hypermetropia, astigmatism	Myopia, astigmatism	+ (type unknown)	Not tested	Hyperopia	–	NR	NR
Strabismus	–	–	–	Not tested	–	NR	NR	NR
Other	Nystagmus	–	–	–	–	NR	NR	Abnormal optic nerve, increased intraocular pressure
**Dental**								
Dental anomalies	–	–	–	Overbite	–	Atypical teeth	Neonatal teeth	NR
**Immune system function**								
Frequent infection	+	–	–	–	–	Low TREC at birth	No TREC at birth	NR
Allergy/asthma	Asthma (light), allergy for HDM and grass pollen	–	–	–	–	+	NR	NR
**Other**								
Other features	• Hypothyroidism • Bicornate uterus	Proportionate short stature with delayed bone age	–	–	One epileptic episode	Mildly dilated aorta, severe obstructive sleep apnea due to micrognathia	Wormian skull bones, multiple brain anomalies on MRI (e.g., absent corpus callosum), umbilical hernia, mild pulmonary artery stenosis spastic quadriplegia and seizures	Scoliosis

*Description of phenotypical and genetic information regarding our patients as well as previously reported patients. The patients reported by Goos et al. (32), Lessel et al. (28), and Punwani et al. (27) carried variants NM_138576.3(BCL11B):c.7C>A, p.(Arg3Ser), NM_138576.3(BCL11B): c.2421C>G, p.(Asn807Lys), and NM_138576.3(BCL11B): c.1323T>G, p.(Asn441Lys), respectively. The patient reported by Longoni et al. (30) carried a variant of BCL11B (BCL11B:NM_138576:exon4:c.C1048T: p.(Arg350Cys)*.

* *, According to NM_138576*;

** *, expression of variant verified in RNA extracted from peripheral blood lymphocyte; HDM, house dust mite, TREC, T-cell-receptor excision circle; NR, not reported*;

‡ *, Combined Annotation Dependent Depletion, GRCh37-V1.6 (33, 34)*.

## Discussion

In this study, we present four new patients with missense variants in *BCL11B* (Patient A: p.(Gly667Glu); Patient B: p. (Gly582Ser); Patient C: p. (Pro673Arg); Patient D: p.(Pro422Leu). Patient A displayed both CDH as well as craniosynostosis of the sagittal suture. The other three patients were diagnosed with craniosynostosis without a diaphragm defect. To our knowledge, this is the second study to report on craniosynostosis in patients with *BCL11B* mutations and the first to report on the co-occurrence of CDH and craniosynostosis in a patient with a missense *BCL11B* variant. Based on our current cohort of patients with BCL11B missense variants, the phenotype associated with *BCL11B* mutations is highly variable. Clinical features range from isolated craniosynostosis to CDH, severe immunological deficiencies and neurodevelopmental delays (Table 1).

*BCL11B* has a key function in fetal development and is involved in a multitude of systems and pathways (35). In line with this, many patients with BCL11B missense mutations are reported to suffer from a wide array of clinical anomalies (27, 28). In addition to altered craniofacial development, mutations, mostly loss of function mutations in BCL11B, have been reported to affect neurodevelopment as well as the development of the immune system, skin and the teeth (27–29, 36–49). In mice models, BCL11B is key in regulating suture patency, with a single disrupted allele causing synostosis (36). Goos et al. further investigated *BCL11B* variants in relation to craniosynostosis (29). A mouse model confirmed that the *de novo BCL11B* missense mutation (p.Arg3Ser) in their patient could have a causative effect on the development of craniosynostosis. Furthermore, they found several rare variants in a British cohort of craniosynostosis patients. However, these variants were dismissed as polymorphisms because of low impact or because they were inherited from a healthy parent. The authors suggest that only a specific subset of *BCL11B* mutations may cause craniosynostosis.

Our patients inherited the *BCL11B* variants from phenotypically normal parents. This could suggest incomplete penetrance, which may be similar to other craniosynostosis syndromes such as TCF12 and SMAD 6-related craniosynostosis and could potentially complicate genetic counseling (32, 50–52). Notably, patients reported in previous *BCL11B* studies mainly were carriers of *de novo* mutations. Although Combined Annotation Dependent Depletion (CADD) scores in our patients vary, patient A, C and D are in the same range as CADD scores of variants previously reported (27–29, 33, 34). Although a low CADD score could potentially indicate a benign variant, it is remarkable that four patients have now presented with craniosynostosis bearing a missense variant in exon 4 of *BCL11B*. Expression of this missense variant in blood excluded non-sense mediated RNA decay in patients A and B. Hence a dominant negative effect or an altered function of the mutated protein could be an explanation of the more severe phenotype.

To our knowledge, only one previous study reported on a patient with a CDH with a *BCL11B* missense mutation [p.(Arg350Cys); CADD score: 31] (30). We now report on a second patient with a *BCL11B* missense variant in exon 4 with CDH. Although further studies remain necessary to assess if *BCL11B* is a causative factor in this CDH phenotype the fact that *BCL11B* is involved in pathways that overlap with pathways that have been previously linked to CDH support this observation. *BCL11B* likely interacts with a CDH causative gene (NR2F2) as well as with other CDH candidate genes (CREBBP, EP300, CHD4) (1, 53). The genetic etiologies of both CDH and craniosynostosis are highly complex and definitive genetic pathways remain to be further elucidated. Although the combination of CDH and craniosynostosis is rare, the fact that multiple syndromes are associated with both craniosynostosis and CDH suggests a possible overlap between craniosynostosis and CDH pathways. For instance, NR2F2, a causative CDH gene with which *BCL11B* has been shown to interact (54, 55), appears to have a function in mesenchymal cell differentiation in embryogenesis including a regulatory function in myogenesis, chondrogenesis, and osteogenesis (56). These processes are disturbed in CDH and craniosynostosis. NR2F2 also regulates Runx2, which has been reported to be overexpressed in some types of craniosynostosis, and has a function in the retinoic acid signaling pathway regulation, which is key in the development of CDH (1, 56–61). Further studies are needed to establish definitive CDH and craniosynostosis pathways and to assess if these pathways are interlinked.

The phenotype of our patients is similar to previously reported clinical phenotypes of patients with *BCL11B* missense mutations, which included craniosynostosis and CDH (6, 27, 28). Therefore, it is implied that both CDH and craniosynostosis may be features associated with *BCL11B* missense mutations. Further functional studies are required to assess if these variants are coincidental findings or if they indeed have a causative effect on craniosynostosis and CDH.

Patients with missense mutations in *BCL11B* appear to be affected more severely than patients with loss of function or other types of *BCL11B* mutations (28). In a previous study, the most severely affected patient carried a missense mutation in a zinc-finger domain in exon 4 (patient EII-I, Table 1) (28). *BCL11B* encodes for a zing finger protein transcription factor and function both as a transcriptional activator as well as a repressor (35, 62). Lessel et al. hypothesized that these missense mutations may not only lead to a loss of DNA binding but also to novel DNA binding sites in other genes (28). Similar observations were made in a previous study, which demonstrated that a mutation p.(Asn441Lys) led to both decreased binding of BCL11B to original target DNA sites as well as to the promotion of novel target DNA binding sites (27). We hypothesize that also these BCL11B missense variants may either have a dominant negative effect or induce new target genes, thereby causing a more severe phenotype in patients with these missense variants /mutations as compared to eg loss of function type of mutations. Future studies should further examine the *in vivo* effect of these specific *BCL11B* mutations in animal models to establish if the described variants are indeed disease causing mutants or coincidental findings, as shown by Goos et al. for the mutation in their patient (29).

Although we are the first to report on the co-occurrence of CDH and craniosynostosis in a patient with a *BCL11B* missense mutation, the co-occurrence of CDH and craniosynostosis has been described previously for several syndromes, such as Apert's syndrome and craniofrontonasal syndrome (7–22). Craniosynostosis syndromes comprise ~30% of all craniosynostosis cases (63). Syndromic craniosynostosis is highly heterogeneous and is often associated with extracranial anomalies, including neurologic, limb, ophthalmologic and cardiac anomalies. Most craniosynostosis syndromes have an autosomal dominant inheritance pattern, although many cases arise from *de novo* mutations (32, 64). We conducted a literature search to identify which craniosynostosis syndromes are associated with CDH. We found nine syndromes to include both CDH as well as craniosynostosis as an associated feature, based on searches in OMIM, Medline, Science Direct and a gray literature search in Google Scholar. Table 2A summarizes the genetic anomalies as well as the main features of each syndrome reported to include both craniosynostosis as well as CDH. In addition, we found seven isolated case reports that describe patients presenting with both craniosynostosis as well as CDH, which are shown in Table 2B. The supplemental information includes Table 2 with a full reference list. Although CDH appears to be a rare feature in craniosynostosis syndromes, craniosynostosis is often treatable with surgical intervention and has high survival rates. In contrast, CDH is a potentially lethal disorder and is associated strongly with poor long-term clinical outcome (66–68). Awareness of this rare feature in craniosynostosis syndromes therefore is key.

**Table 2A:** Overview of disorders characterized by co-occurrence of craniosynostosis and congenital diaphragmatic hernia.

**Clinical disorders with autosomal dominant inheritance pattern**							
	**Gene (MIM number)**	**Phenotype MIM number**	**Chromosome**	CS*	**CDH^‡^**	**Key clinical features**	**Authors reporting on CDH and/or CS**
Apert syndrome	FGFR2 (176943)	101200	10q26.13	Key feature, multisutural, progressive	Rare (six cases of CDH and one case of diaphragm agenesis)	– Symmetric syndactyly of hands and feet – Midface hypoplasia	Kaur 2019 Dap 2019 Kosinski 2016 Sobaih 2015 Bulfamante 2011 Wallis-Crespo 2004 Witters 2000
Kabuki syndrome (focus on type 1)	KMT2D (602113)	147920	12q13.12	Occasional	Relatively common	– Characteristic facial features: long palpebral fissures, everted lower eyelids, ptosis, arched eyebrows, blue sclera, cupped ears, micrognathia – Short stature, microcephaly – Intellectual disability (mild to moderate) – High/cleft palate and dental anomalies – Brachydactyly, clinodactyly, persistent fetal pads – Cardiac anomalies	Scott 2021 Topa 2017 Martinez-Lopez 2010 David 2004 Geneviève 2004 Van Haelst 2000
CEBALID** syndrome (MN1 C-terminal truncation syndrome)	MN1 (156100)	618774	22q12.1	Reported in three patients out of 25 identified patients identified to date	Reported in two patients out of 25 identified patients identified to date	– Characteristic facial features: midface hypoplasia, downslanting palpebral fissures, hypertelorism, exophthalmia, low-set ears, a short upturned nose – Intellectual disability, hypotonia, delay in motor development – Hearing loss – Structural brain anomalies	Mak 2020
Chromosome 22q11.2 deletion syndrome	-	145410; 188400; 192430; 600594; 601279; 601755; 602054; 609030	22q11.2	Rare feature (*may include CDC45 pathogenic variant in remaining allele*	Rare feature	– Highly variable phenotype (ranging from minor abnormities to major structural defects) – Cardiovascular anomalies – Cleft palate – Cognitive impairment – Short stature – Characteristic facial features: hypoplastic nasal alae, wide nasal bridge, short palpebral fissures, low-set, small ears – Nasal speech	Unolt 2020, 2017 McDonal-McGinn 2005
SPECC1L- related syndromes	SPECC1L (614140)	145410 145420 600251	22q11.23	Occasional	Occasional occurrence	– Characteristic facial features: hypertelorism, a wide, short nose, ptosis and retrognathia – Cleft lip/palate – Clinical features include branchial fistulas, omphalocele, genitourinary anomalies	Wild 2020 Bhoj 2019 Kruszka 2015 Robin 1995
7q11. 23 Duplication syndrome	-	609757	7q11.23	Rare	Rare	– Variable expression, with incomplete penetrance – Characteristic facial features: prominent forehead, hypertelorism, high and broad nose, straight eyebrows, and thin lips – Cognitive impairment and intellectual disability – Epilepsy	Morris 2015 Van der Aa 2009 Torniero 2008 Kriek 2006
**X-Linked**												
Craniofrontonasal syndrome (XLD)	EFNB1 (300035)	304110	Xq13.1	Common feature, often either unilateral or bilateral coronal CS	Relatively common/ occasional	– More severe phenotype in females – Characteristic facial features: hypertelorism, craniofacial asymmetry, webbed neck, bifid tip of the nose, a broad nasal bridge – Clinodactyly of ≥ 1 digit – Longitudinal splitting/ridging of nails	Hogue 2010 Kawamoto 2007 Vasudevan 2006 Twigg 2004 & 2006 Brooks 2002 McGaughran 2002 Hurst 1988 Morris 1987
Cornelia de Lange syndrome	NIPBL (608667, AD) SMC1A (300040, XLD)	122470 300590	5p13.2 Xp11.22	Described for NIPBL variant; and for SMCA1	Key feature	– Characteristic facial features: thick, arched eyebrows or synophrys, long/smooth philtrum, short nose, thin upper vermillion – Limb defects – Intellectual disability – Growth retardation – Hirsutism	Desai 2021 Xu 2018 Gupta 2020
Simpson-Golabi-Behmel syndrome, Type 1 (XLR)	GPC3 (300037)	312870	Xq26.2	3 case reports	Occasional	– Characteristic facial features: hypertelorism, downslanting palpebral fissures – Cleft palate/lip. – Overgrowth and macrocephaly – Intellectual disability – Cardiac anomalies – Renal abnormality – Brachy-, syn-, and polydactyly	Schirwani 2019 Villarreal 2013 Li 2009

*This table presents an overview of clinical disorders in which both craniosynostosis and congenital diaphragmatic hernia have been reported more than once*.

* *CS, craniosynostosis*;

‡ *CDH, congenital diaphragmatic hernia*;

** *CEBALID, craniofacial defects, dysmorphic ears, structural brain abnormalities, expressive language delay, and impaired intellectual development; XLD, X-linked dominant; XLR, X-linked recessive*.

**Table 2B:** Isolated case reports on the co-occurrence of craniosynostosis and congenital diaphragmatic hernia.

	**Gene (MIM number)**	**Phenotype** **MIM number**	**Chromosome**	CS*	**CDH^‡^**	**Key clinical features**	**Authors reporting on CDH and/or CS**
Saethre -Chotzen	TWIST1 (601622)	101400	7p21.1	Key feature, often bicoronal CS	One case, unclear if co-occurrence of CDH is coincidental. Mouse models suggest a possible role for TWIST1 in development of the diaphragm	– Characteristic facial features: ptosis, downward slanting palpebral fissure, depressed nasal bridge, facial asymmetry – Small ears with prominent crus – Syndactyly of hand and feet	Piard 2012
Chromosome 9p deletion syndrome	-	158170	9p	Key feature: metopic CS	One case described for 9p deletion syndrome	– Characteristic facial features: hypotelorism, upslanting palpebral fissures, low-set ears, malformed ears, long philtrum – Moderate to severe intellectual disability	Alfi 1973
15q24 deletion syndrome	-	613406	15q24.2	1 case report	Four reports	– Characteristic facial features: high forehead, facial asymmetry, downslanting of eyes, hypertelorism, and a long smooth philtrum, ear malformations – Intellectual disability – Genitourinary anomalies – Cardiovascular malformations	Ng 2011 Van Esch 2009 Sharp 2007 Bettelheim 1998
DPF2-related Coffin–Siris syndrome	DPF2 (601671)	618027	11q12.1	At least two out of a total of 10 reported patients (one patient was stated to have trigonocephaly but no x-ray was performed) b	One patient described out of a total of 10 reported patients.	– Cognitive impairment, intellectual disability, and behavioral problems – Feeding problems and hypotonia – Hearing loss – Brachydactyly, clinodactyly, hypoplastic nails – Coarse facial features	Knapp 2019 Vasileiou 2018
-	DSC2 (125645)	-		One report of a patient with multisutural CS and CDH	One report of a patient with multisutural CS and CDH	Isolated case: presented with left atrial isomerism, transposed systemic and pulmonary veins, intestinal malrotation, bilateral inguinal hernia, hydronephrosis and nephrolithiasis in addition to CDH and CS	Das 2019
Loeys-Dietz syndrome	TGFBR1 (190181) TGFBR2 (190182)	609192 610168	9q22.33 3p24.1	Multiple cases reported	One report	– Aortic and arterial aneurysms – Characterstic facial features: hypertelorism, downslant of the eyes – Cleft palate, bifid uvula – Pectus anomalies – Arachnodactyly	Lobaton 2021 Loeys 2005
Gain of function of RARB	RARB			One report of a patient with CS	Multiple patients with diaphragmatic hernia	Thirteen cases have been reported in total. Clinical features include microphthalmia and anophthalmia, sclerocornea, and coloboma, as well as cardiac anomalies, and malrotation of the bowel	Srour 2016

* *CS, craniosynostosis*;

‡ *CDH, congenital diaphragmatic hernia*.

## Conclusion

This report implies that both CDH as well as craniosynostosis are features of *BCL11B* missense mutations. However, further studies are required to establish if *BCL11B* missense mutations are indeed a causative factor or if our finding was coincidental.

## Data Availability Statement

The original contributions presented in the study are included in the article/Supplementary Material, further inquiries can be directed to the corresponding author/s.

## Ethics Statement

Ethical review and approval was not required for the study on human participants in accordance with the local legislation and institutional requirements. Written informed consent to participate in this study was provided by the participants' legal guardian/next of kin. Written informed consent was obtained from the individual(s), and minor(s)' legal guardian/next of kin, for the publication of any potentially identifiable images or data included in this article.

## Author Contributions

LG, AK, IM, and MD contributed to the study's conception, wrote the manuscript, and provided critical revisions. LG, AG, and MD assessed the clinical and phenotypical features of the patients. FM and FJ assisted with data collection and provided genetic information. AG, QB, DV, and EB provided critical revision. All authors approved the article for submission.

## Conflict of Interest

The authors declare that the research was conducted in the absence of any commercial or financial relationships that could be construed as a potential conflict of interest.

## Publisher's Note

All claims expressed in this article are solely those of the authors and do not necessarily represent those of their affiliated organizations, or those of the publisher, the editors and the reviewers. Any product that may be evaluated in this article, or claim that may be made by its manufacturer, is not guaranteed or endorsed by the publisher.

## References

[1] Kammoun M Souche E Brady P Ding J Cosemans N Gratacos E. Genetic profile of isolated congenital diaphragmatic hernia revealed by targeted next-generation sequencing. Prenat Diagn. (2018) 38:654–63. 10.1002/pd.5327 29966037

[2] McGivern MR Best KE Rankin J Wellesley D Greenlees R Addor MC. Epidemiology of congenital diaphragmatic hernia in Europe: a register-based study. Arch Dis Child Fetal Neonatal Ed. (2015) 100:F137–44. 10.1136/archdischild-2014-306174 25411443

[3] Gallot D Boda C Ughetto S Perthus I Robert-Gnansia E Francannet C. Prenatal detection and outcome of congenital diaphragmatic hernia: a French registry-based study. Ultrasound Obstet Gynecol. (2007) 29:276–83. 10.1002/uog.3863 17177265

[4] Paoletti M Raffler G Gaffi MS Antounians L Lauriti G Zani A. Prevalence and risk factors for congenital diaphragmatic hernia: a global view. J Pediatr Surg. (2020) 55:2297–307. 10.1016/j.jpedsurg.2020.06.022 32690291

[5] Zaiss I Kehl S Link K Neff W Schaible T Sutterlin M. Associated malformations in congenital diaphragmatic hernia. Am J Perinatol. (2011) 28:211–8. 10.1055/s-0030-1268235 20979012

[6] Robert E Kallen B Harris J. The epidemiology of diaphragmatic hernia. Eur J Epidemiol. (1997) 13:665–73. 10.1023/A:1007395727819 9324213

[7] Kaur R Mishra P Kumar S Sankar MJ Kabra M Gupta N. Apert syndrome with congenital diaphragmatic hernia: another case report and review of the literature. Clin Dysmorphol. (2019) 28:78–80. 10.1097/MCD.0000000000000261 30672749

[8] Bulfamante G Gana S Avagliano L Fabietti I Gentilin B Lalatta F. Congenital diaphragmatic hernia as prenatal presentation of Apert syndrome. Prenat Diagn. (2011) 31:910–1. 10.1002/pd.2788 21706505

[9] Dap M Bach-Segura P Bertholdt C Menzies D Masutti JP Klein O. Variable phenotypic expression of Apert syndrome in monozygotic twins. Clin Case Rep. (2019) 7:54–7. 10.1002/ccr3.1915 30656008 PMC6333066

[10] Kosinski P Luterek K Wielgos M. Diaphragmatic hernia as an early ultrasound manifestation of Apert syndrome. Ginekol Pol. (2016) 87:830. 10.5603/GP.2016.0097 28098935

[11] Sobaih BH AlAli AA. A third report of Apert syndrome in association with diaphragmatic hernia. Clin Dysmorphol. (2015) 24:106–8. 10.1097/MCD.0000000000000083 25714562

[12] Wallis-Crespo MC Gilbert-Barness E. Pathology teach and tell: acrocephalosyndactyly type I (Apert syndrome). Fetal Pediatr Pathol. (2004) 23:71–8. 10.1080/15227950490423160 15371125

[13] Witters I Devriendt K Moerman P van Hole C Fryns JP. Diaphragmatic hernia as the first echographic sign in Apert syndrome. Prenat Diagn. (2000) 20:404–6. 10.1002/(SICI)1097-0223(200005)20:5<404::AID-PD813>3.0.CO;2-0 10820409

[14] Brooks AS van Dooren M Hoogeboom J Gischler S Willems PJ Tibboel D. Congenital diaphragmatic hernia in a female patient with craniofrontonasal syndrome. Clin Dysmorphol. (2002) 11:151–3. 10.1097/00019605-200204000-00019 12002152

[15] Hogue J Shankar S Perry H Patel R Vargervik K Slavotinek A. A novel EFNB1 mutation (c.712delG) in a family with craniofrontonasal syndrome and diaphragmatic hernia. Am J Med Genet Part A. (2010) 152A:2574–7. 10.1002/ajmg.a.33596 20734337

[16] Hurst J Baraitser M. Craniofrontonasal dysplasia. J Med Genet. (1988) 25:133–4. 10.1136/jmg.25.2.133 3346887 PMC1015457

[17] Kawamoto HK Heller JB Heller MM Urrego A Gabbay JS Wasson KL. Craniofrontonasal dysplasia: a surgical treatment algorithm. Plast Reconstr Surg. (2007) 120:1943–56. 10.1097/01.prs.0000287286.12944.9f 18090758

[18] McGaughran J Rees M Battin M. Craniofrontonasal syndrome and diaphragmatic hernia. Am J Med Genet. (2002) 110:391–2. 10.1002/ajmg.10176 12116215

[19] Morris CA Palumbos JC Carey JC. Delineation of the male phenotype in carniofrontonasal syndrome. Am J Med Genet. (1987) 27:623–31. 10.1002/ajmg.1320270315 3631134

[20] Twigg SR Kan R Babbs C Bochukova EG Robertson SP Wall SA. Mutations of ephrin-B1 (EFNB1), a marker of tissue boundary formation, cause craniofrontonasal syndrome. Proc Natl Acad Sci USA. (2004) 101:8652–7. 10.1073/pnas.0402819101 15166289 PMC423250

[21] Twigg SR Matsumoto K Kidd AM Goriely A Taylor IB Fisher RB. The origin of EFNB1 mutations in craniofrontonasal syndrome: frequent somatic mosaicism and explanation of the paucity of carrier males. Am J Hum Genet. (2006) 78:999–1010. 10.1086/504440 16685650 PMC1474108

[22] Vasudevan PC Twigg SR Mulliken JB Cook JA Quarrell OW Wilkie AO. Expanding the phenotype of craniofrontonasal syndrome: two unrelated boys with EFNB1 mutations and congenital diaphragmatic hernia. Eur J Hum Genet. (2006) 14:884–7. 10.1038/sj.ejhg.5201633 16639408

[23] Cornelissen M Ottelander B Rizopoulos D van der Hulst R Mink van der Molen A van der Horst C. Increase of prevalence of craniosynostosis. J Craniomaxillofac Surg. (2016) 44:1273–9. 10.1016/j.jcms.2016.07.007 27499511

[24] de Jong T Bannink N Bredero-Boelhouwer HH van Veelen ML Bartels MC Hoeve LJ. Long-term functional outcome in 167 patients with syndromic craniosynostosis; defining a syndrome-specific risk profile. J Plast Reconstr Aesthet Surg. (2010) 63:1635–41. 10.1016/j.bjps.2009.10.029 19913472

[25] Maliepaard M Mathijssen IM Oosterlaan J Okkerse JM. Intellectual, behavioral, and emotional functioning in children with syndromic craniosynostosis. Pediatrics. (2014) 133:e1608–15. 10.1542/peds.2013-3077 24864183

[26] Mathijssen IMJ Craniosynostosis Working Group. Guideline. Updated guideline on treatment and management of craniosynostosis. J Craniofac Surg. (2021) 32:371–450. 10.1097/SCS.0000000000007035 33156164 PMC7769187

[27] Punwani D Zhang Y Yu J Cowan MJ Rana S Kwan A. Multisystem anomalies in severe combined immunodeficiency with mutant BCL11B. N Engl J Med. (2016) 375:2165–76. 10.1056/NEJMoa1509164 27959755 PMC5215776

[28] Lessel D Gehbauer C Bramswig NC Schluth-Bolard C Venkataramanappa S van Gassen KLI. BCL11B mutations in patients affected by a neurodevelopmental disorder with reduced type 2 innate lymphoid cells. Brain. (2018) 141:2299–311. 10.1093/brain/awy173 29985992 PMC6061686

[29] Goos JAC Vogel WK Mlcochova H Millard CJ Esfandiari E Selman WH. A *de novo* substitution in BCL11B leads to loss of interaction with transcriptional complexes and craniosynostosis. Hum Mol Genet. (2019) 28:2501–13. 10.1093/hmg/ddz072 31067316 PMC6644156

[30] Longoni M High FA Qi H Joy MP Hila R Coletti CM. Genome-wide enrichment of damaging *de novo* variants in patients with isolated and complex congenital diaphragmatic hernia. Hum Genet. (2017) 136:679–91. 10.1007/s00439-017-1774-y 28303347 PMC5453716

[31] Tweedie S Braschi B Gray K Jones TEM Seal RL Yates B. Genenames.org: the HGNC and VGNC resources in 2021. Nucleic Acids Res. (2021) 49:D939–46. 10.1093/nar/gkaa980 33152070 PMC7779007

[32] Goos JAC Mathijssen IMJ. Genetic causes of craniosynostosis: an update. Mol Syndromol. (2019) 10:6–23. 10.1159/000492266 30976276 PMC6422124

[33] CADD. University of Washington Hudson-Alpha Institute for Biotechnology and Berlin Institute of Health. Combined Annotation Dependent Depletion; Single Nucleotide Variant (SNV) Lookup. (2021). Available online at: https://cadd.gs.washington.edu/snv

[34] Rentzsch P Schubach M Shendure J Kircher M. CADD-Splice-improving genome-wide variant effect prediction using deep learning-derived splice scores. Genome Med. (2021) 13:31. 10.1186/s13073-021-00835-9 33618777 PMC7901104

[35] Lennon MJ Jones SP Lovelace MD Guillemin GJ Brew BJ. Bcl11b-A critical neurodevelopmental transcription factor-roles in health and disease. Front Cell Neurosci. (2017) 11:89. 10.3389/fncel.2017.00089 28424591 PMC5372781

[36] Kyrylkova K Iwaniec UT Philbrick KA Leid M. BCL11B regulates sutural patency in the mouse craniofacial skeleton. Dev Biol. (2016) 415:251–60. 10.1016/j.ydbio.2015.10.010 26453795 PMC4826646

[37] Wakabayashi Y Watanabe H Inoue J Takeda N Sakata J Mishima Y. Bcl11b is required for differentiation and survival of alphabeta T lymphocytes. Nat Immunol. (2003) 4:533–9. 10.1038/ni927 12717433

[38] Li L Leid M Rothenberg EV. An early T cell lineage commitment checkpoint dependent on the transcription factor Bcl11b. Science. (2010) 329:89–93. 10.1126/science.1188989 20595614 PMC2935300

[39] Albu DI VanValkenburgh J Morin N Califano D Jenkins NA Copeland NG. Transcription factor Bcl11b controls selection of invariant natural killer T-cells by regulating glycolipid presentation in double-positive thymocytes. Proc Natl Acad Sci USA. (2011) 108:6211–6. 10.1073/pnas.1014304108 21444811 PMC3076841

[40] Arlotta P Molyneaux BJ Jabaudon D Yoshida Y Macklis JD. Ctip2 controls the differentiation of medium spiny neurons and the establishment of the cellular architecture of the striatum. J Neurosci. (2008) 28:622–32. 10.1523/JNEUROSCI.2986-07.2008 18199763 PMC6670353

[41] Golonzhka O Liang X Messaddeq N Bornert JM Campbell AL Metzger D. Dual role of COUP-TF-interacting protein 2 in epidermal homeostasis and permeability barrier formation. J Invest Dermatol. (2009) 129:1459–70. 10.1038/jid.2008.392 19092943 PMC2754722

[42] Golonzhka O Metzger D Bornert JM Bay BK Gross MK Kioussi C. Ctip2/Bcl11b controls ameloblast formation during mammalian odontogenesis. Proc Natl Acad Sci USA. (2009) 106:4278–83. 10.1073/pnas.0900568106 19251658 PMC2657370

[43] Kastner P Chan S Vogel WK Zhang LJ Topark-Ngarm A Golonzhka O. Bcl11b represses a mature T-cell gene expression program in immature CD4(+)CD8(+) thymocytes. Eur J Immunol. (2010) 40:2143–54. 10.1002/eji.200940258 20544728 PMC2942964

[44] Simon R Baumann L Fischer J Seigfried FA De Bruyckere E Liu P. Structure-function integrity of the adult hippocampus depends on the transcription factor Bcl11b/Ctip2. Genes Brain Behav. (2016) 15:405–19. 10.1111/gbb.12287 26915960 PMC4832350

[45] Simon R Brylka H Schwegler H Venkataramanappa S Andratschke J Wiegreffe C. A dual function of Bcl11b/Ctip2 in hippocampal neurogenesis. EMBO J. (2012) 31:2922–36. 10.1038/emboj.2012.142 22588081 PMC3395096

[46] Uddin MN Zhang Y Harton JA MacNamara KC Avram D. TNF-alpha-dependent hematopoiesis following Bcl11b deletion in T cells restricts metastatic melanoma. J Immunol. (2014) 192:1946–53. 10.4049/jimmunol.1301976 24446520 PMC3946214

[47] Vanvalkenburgh J Albu DI Bapanpally C Casanova S Califano D Jones DM. Critical role of Bcl11b in suppressor function of T regulatory cells and prevention of inflammatory bowel disease. J Exp Med. (2011) 208:2069–81. 10.1084/jem.20102683 21875956 PMC3182057

[48] Kyrylkova K Kyryachenko S Biehs B Klein O Kioussi C Leid M. BCL11B regulates epithelial proliferation and asymmetric development of the mouse mandibular incisor. PLoS ONE. (2012) 7:e37670. 10.1371/journal.pone.0037670 22629441 PMC3358280

[49] Albu DI Feng D Bhattacharya D Jenkins NA Copeland NG Liu P. BCL11B is required for positive selection and survival of double-positive thymocytes. J Exp Med. (2007) 204:3003–15. 10.1084/jem.20070863 17998389 PMC2118514

[50] Timberlake AT Choi J Zaidi S Lu Q Nelson-Williams C Brooks ED. Two locus inheritance of non-syndromic midline craniosynostosis via rare SMAD6 and common BMP2 alleles. Elife. (2016) 5:e20125. 10.7554/eLife.20125 27606499 PMC5045293

[51] Sharma VP Fenwick AL Brockop MS McGowan SJ Goos JA Hoogeboom AJ. Mutations in TCF12, encoding a basic helix-loop-helix partner of TWIST1, are a frequent cause of coronal craniosynostosis. Nat Genet. (2013) 45:304–7. 10.1016/S0140-6736(13)60554-1 23354436 PMC3647333

[52] di Rocco F Baujat G Arnaud E Renier D Laplanche JL Daire VC. Clinical spectrum and outcomes in families with coronal synostosis and TCF12 mutations. Eur J Hum Genet. (2014) 22:1413–6. 10.1038/ejhg.2014.57 24736737 PMC4231413

[53] Szklarczyk D Gable AL Lyon D Junge A Wyder S Huerta-Cepas J. STRING v11: protein-protein association networks with increased coverage, supporting functional discovery in genome-wide experimental datasets. Nucleic Acids Res. (2019) 47:D607–13. 10.1093/nar/gky1131 30476243 PMC6323986

[54] Sidwell T Rothenberg EV. Epigenetic dynamics in the function of t-lineage regulatory factor Bcl11b. Front Immunol. (2021) 12:669498. 10.3389/fimmu.2021.669498 33936112 PMC8079813

[55] Avram D Fields A Pretty On Top K Nevrivy DJ Ishmael JE Leid M. Isolation of a novel family of C(2)H(2) zinc finger proteins implicated in transcriptional repression mediated by chicken ovalbumin upstream promoter transcription factor (COUP-TF) orphan nuclear receptors. J Biol Chem. (2000) 275:10315–22. 10.1074/jbc.275.14.10315 10744719 PMC2819356

[56] Xie X Qin J Lin SH Tsai SY Tsai MJ. Nuclear receptor chicken ovalbumin upstream promoter-transcription factor II (COUP-TFII) modulates mesenchymal cell commitment and differentiation. Proc Natl Acad Sci USA. (2011) 108:14843–8. 10.1073/pnas.1110236108 21873211 PMC3169151

[57] Cuellar A Bala K Di Pietro L Barba M Yagnik G Liu JL. Gain-of-function variants and overexpression of RUNX2 in patients with nonsyndromic midline craniosynostosis. Bone. (2020) 137:115395. 10.1016/j.bone.2020.115395 32360898 PMC7358991

[58] Lee KN Jang WG Kim EJ Oh SH Son HJ Kim SH. Orphan nuclear receptor chicken ovalbumin upstream promoter-transcription factor II (COUP-TFII) protein negatively regulates bone morphogenetic protein 2-induced osteoblast differentiation through suppressing runt-related gene 2 (Runx2) activity. J Biol Chem. (2012) 287:18888–99. 10.1074/jbc.M111.311878 22493443 PMC3365924

[59] Kruse SW Suino-Powell K Zhou XE Kretschman JE Reynolds R Vonrhein C. Identification of COUP-TFII orphan nuclear receptor as a retinoic acid-activated receptor. PLoS Biol. (2008) 6:e227. 10.1371/journal.pbio.0060227 18798693 PMC2535662

[60] Kardon G Ackerman KG McCulley DJ Shen Y Wynn J Shang L. Congenital diaphragmatic hernias: from genes to mechanisms to therapies. Dis Model Mech. (2017) 10:955–70. 10.1242/dmm.028365 28768736 PMC5560060

[61] Clugston RD Zhang W Alvarez S de Lera AR Greer JJ. Understanding abnormal retinoid signaling as a causative mechanism in congenital diaphragmatic hernia. Am J Respir Cell Mol Biol. (2010) 42:276–85. 10.1165/rcmb.2009-0076OC 19448158

[62] Kominami R. Role of the transcription factor Bcl11b in development and lymphomagenesis. Proc Jpn Acad Ser B Phys Biol Sci. (2012) 88:72–87. 10.2183/pjab.88.72 22450536 PMC3365246

[63] Wilkie AOM Johnson D Wall SA. Clinical genetics of craniosynostosis. Curr Opin Pediatr. (2017) 29:622–8. 10.1097/MOP.0000000000000542 28914635 PMC5681249

[64] Twigg SR Wilkie AO. A genetic-pathophysiological framework for craniosynostosis. Am J Hum Genet. (2015) 97:359–77. 10.1016/j.ajhg.2015.07.006 26340332 PMC4564941

[65] Srour M Caron V Pearson T Nielsen SB Levesque S Delrue MA. Gain-of-function mutations in RARB cause intellectual disability with progressive motor impairment. Hum Mutat. (2016) 37:786–93. 10.1002/humu.23004 27120018

[66] Congenital Diaphragmatic Hernia Study G Lally KP Lally PA Lasky RE Tibboel D Jaksic T. Defect size determines survival in infants with congenital diaphragmatic hernia. Pediatrics. (2007) 120:e651–7. 10.1542/peds.2006-3040 17766505

[67] Coughlin MA Werner NL Gajarski R Gadepalli S Hirschl R Barks J. Prenatally diagnosed severe CDH: mortality and morbidity remain high. J Pediatr Surg. (2016) 51:1091–5. 10.1016/j.jpedsurg.2015.10.082 26655216

[68] Chatterjee D Ing RJ Gien J. Update on congenital diaphragmatic hernia. Anesth Analg. (2020) 131:808–21. 10.1213/ANE.0000000000004324 31335403

