# Supplementary material for: Case Report and Review of the Literature: Congenital Diaphragmatic Hernia and Craniosynostosis, a Coincidence or Common Cause?
Source: Front Pediatr. 2021 Nov 26;9:772800. doi: 10.3389/fped.2021.772800 (PMC8662985; doi:10.3389/fped.2021.772800)
Supplement: Supplementary file 1 [file Table_1.docx]

| **Supplemental Table 1A. Overview of disorders characterized by co-occurrence of craniosynostosis and congenital diaphragmatic hernia**  **Clinical disorders with autosomal dominant inheritance pattern** | | | | | | |  |  |
| --- | --- | --- | --- | --- | --- | --- | --- | --- |
|  | **Gene**  **(MIM number)** | **Phenotype MIM number** | **chromosome** | **CS*** | **CDH^‡^** | **Key clinical features** | **Authors reporting on CDH and/or CS** |  |
| **Apert syndrome** | FGFR2 (176943) | 101200 | 10q26.13 | Key feature, multisutural, progressive | Rare  (six cases of CDH (1-6) and one case of diaphragm agenesis(7)) | - Symmetric syndactyly of hands and feet - Midface hypoplasia(8) | Kaur2019  Dap2019  Kosinski2016  Sobaih2015  Bulfamante2011  Wallis-Crespo2004  Witters2000 |  |
| **Kabuki syndrome (focus on type 1)** | KMT2D (602113) | 147920 | 12q13.12 | Occasional(9-11) | Relatively common(12-15) | - Characteristic facial features: long palpebral fissures, everted lower eyelids, ptosis, arched eyebrows, blue sclera, cupped ears, micrognathia - Short stature, microcephaly - Intellectual disability (mild to moderate) - High/cleft palate and dental anomalies - Brachydactyly, clinodactyly, persistent fetal pads - Cardiac anomalies (16) | Scott2021  Topa2017  Martinez-Lopez2010  David2004  Geneviève2004  Van Haelst2000 |  |
| **CEBALID** syndrome**  **(MN1 C-terminal truncation syndrome)** | MN1  (156100) | 618774 | 22q12.1 | Reported in three patients out of 25 identified patients identified to date(17, 18) | Reported in two patients out of 25 identified patients identified to date (17, 18) | - Characteristic facial features: midface hypoplasia, downslanting palpebral fissures, hypertelorism, exophthalmia, low-set ears, a short upturned nose - Intellectual disability, hypotonia, delay in motor development - Hearing loss - Structural brain anomalies(17, 18) | Mak 2020 |  |
| **Chromosome 22q11.2 deletion syndrome** |  | 145410; 188400; 192430; 600594; 601279; 601755; 602054; 609030 | 22q11.2 | Rare feature(19)  (*may include CDC45 pathogenic variant in remaining allele*(20)) | Rare feature(21) | - Highly variable phenotype (ranging from minor abnormities to major structural defects) - Cardiovascular anomalies - Cleft palate - Cognitive impairment - Short stature - Characteristic facial features: hypoplastic nasal alae, wide nasal bridge, short palpebral fissures,   low-set, small ears   - Nasal speech(22, 23) | Unolt 2020, 2017  McDonal-McGinn 2005 |  |
| **SPECC1L- related syndromes** | SPECC1L (614140) | 145410  145420 600251 | 22q11.23 | Occasional(24) | Occasional occurrence(24-27) | - Characteristic facial features: hypertelorism, a wide, short nose, ptosis and retrognathia - Cleft lip/palate - Clinical features include branchial fistulas, omphalocele, genitourinary anomalies(28) | Wild 2020  Bhoj 2019  Kruszka 2015  Robin 1995 |  |
| **7q11. 23 Duplication syndrome** | - | 609757 | 7q11.23 | Rare(29-31) | Rare(29, 32) | - Variable expression, with incomplete penetrance - Characteristic facial features: prominent forehead, hypertelorism, high and broad nose, straight eyebrows, and thin lips - Cognitive impairment and intellectual disability - Epilepsy(33) | Morris 2015  Van der Aa 2009  Torniero 2008  Kriek 2006 |  |
| **X-Linked** | |  |  |  |  |  | | |
| **Craniofrontonasal syndrome (XLD)** | EFNB1 (300035) | 304110 | Xq13.1 | Common feature, often either unilateral or bilateral coronal CS(34, 35) | Relatively common/ occasional(36-44) | - More severe phenotype in females - Characteristic facial features: hypertelorism, craniofacial asymmetry, webbed neck, bifid tip of the nose, a broad nasal bridge - clinodactyly of ≥ 1 digit - longitudinal splitting/ridging of nails(35) | Hogue 2010  Kawamoto 2007  Vasudevan 2006  Twigg 2004 & 2006  Brooks 2002  McGaughran2002  Hurst 1988  Morris 1987 |  |
| **Cornelia de Lange syndrome** | NIPBL (608667, AD)  SMC1A  (300040, XLD) | 122470  300590 | 5p13.2  Xp11.22 | Described for NIPBL variant(45); and for SMCA1(46) | Key feature (47) | - Characteristic facial features: thick, arched eyebrows or synophrys, long/smooth philtrum, short nose, thin upper vermillion - Limb defects - Intellectual disability - Growth retardation - Hirsutism (48) | Desai 2021  Xu 2018  Gupta 2020 |  |
| **Simpson-Golabi-Behmel syndrome, Type 1**  **(XLR)** | GPC3(300037) | 312870 | Xq26.2 | 3 case reports(49-51) | Occasional(49, 52) | - Characteristic facial features: hypertelorism, downslanting palpebral fissures - Cleft palate/lip. - Overgrowth and macrocephaly - Intellectual disability - Cardiac anomalies - Renal abnormality, - Brachy-, syn-, and polydactyly.(53) | Schirwani 2019  Villarreal 2013  Li 2009 |  |

**Supplemental Table 1A . Overview of disorders characterized by co-occurrence of craniosynostosis and congenital diaphragmatic hernia.** This table is the supplemental version of Table 2A in the article. It presents an overview of clinical disorders in which both craniosynostosis and congenital diaphragmatic hernia have been reported more than once. Abbreviations: *CS= craniosynostosis, ^‡^CDH = congenital diaphragmatic hernia, ** CEBALID= craniofacial defects, dysmorphic ears, structural brain abnormalities, expressive language delay, and impaired intellectual development, XLD= X-linked dominant, XLR= X-linked recessive.

| **Supplemental Table 1B. Isolated case reports on the co-occurrence of craniosynostosis and congenital diaphragmatic hernia** | | | | | | |  |
| --- | --- | --- | --- | --- | --- | --- | --- |
|  | **Gene**  **(MIM number)** | **Phenotype MIM number** | **chromosome** | **CS*** | **CDH^‡^** | **Key clinical features** | **Authors reporting on CDH and/or CS** |
| **Saethre -Chotzen** | TWIST1  (601622) | 101400 | 7p21.1 | Key feature, often bicoronal CS | One case(54), unclear if co-occurrence of CDH is coincidental. Mouse models suggest a possible role for TWIST1 in development of the diaphragm | - Characteristic facial features: ptosis, downward slanting palpebral fissure, depressed nasal bridge, facial asymmetry - Small ears with prominent crus - Syndactyly of hand and feet (55) | Piard 2012 |
| **Chromosome 9p deletion syndrome** | - | 158170 | 9p | Key feature: metopic CS | One case described for 9p deletion syndrome(56) | - Characteristic facial features: hypotelorism, upslanting palpebral fissures, low-set ears, malformed ears, long philtrum - Moderate to severe intellectual disability | Alfi 1973 |
| **15q24 deletion syndrome** | - | 613406 | 15q24.2 | 1 case report(57) | Four reports (58-60) | - Characteristic facial features: high forehead, facial asymmetry, downslanting of eyes, hypertelorism, and a long smooth philtrum, ear malformations - Intellectual disability - Genitourinary anomalies - Cardiovascular malformations(61, 62) | Ng 2011  Van Esch 2009  Sharp 2007  Bettelheim 1998 |
| **DPF2-related Coffin–Siris syndrome** | DPF2  (601671) | 618027 | 11q12.1 | At least two out of a total of 10 reported patients  (one patient was stated to have trigonocephaly but no x-ray was performed)(63) | One patient described out of a total of 10 reported patients.(64) | - Cognitive impairment, intellectual disability, and behavioral problems - Feeding problems and hypotonia - Hearing loss - Brachydactyly, clinodactyly, hypoplastic nails - Coarse facial features | Knapp 2019  Vasileiou 2018 |
| - | DSC2  (125645) | - |  | One report of a patient with multisutural CS and CDH (65) | One report of a patient with multisutural CS and CDH (65) | Isolated case: presented with left atrial isomerism, transposed systemic and pulmonary veins, intestinal malrotation, bilateral inguinal hernia, hydronephrosis and nephrolithiasis in addition to CDH and CS (65) | Das 2019 |
| **Loeys-Dietz syndrome** | TGFBR1  (190181)  TGFBR2  (190182) | 609192  610168 | 9q22.33  3p24.1 | Multiple cases reported (66) | One report(67) | - Aortic and arterial aneurysms - Characterstic facial features: hypertelorism, downslant of the eyes - Cleft palate, bifid uvula. - Pectus anomalies - Arachnodactyly (66) | Lobaton 2021  Loeys 2005 |
| **Gain of function of RARB** | RARB |  |  | One report of a patient with CS(68) | Multiple patients with diaphragmatic hernia(68, 69) | Thirteen cases have been reported in total. Clinical features include microphthalmia and anophthalmia, sclerocornea, and coloboma, as well as cardiac anomalies, and malrotation of the bowel (68, 69) | Srour 2016 |

**Supplemental Table 1B. Isolated case reports on the co-occurrence of craniosynostosis and congenital diaphragmatic hernia.** This table is the supplemental version of Table 2B in the article. Abbreviations: *CS = craniosynostosis, ^‡^CDH = congenital diaphragmatic hernia.

Mutations in FRAS-related extracellular matrix protein 1 (FREM1, MIM number: 608944) are not shown in this table as reports on this gene are contradictory. Vissers et al suggested CS is a feature of heterozygous FREM1 mutations,(70) although this finding was not supported by Dawson et al (71). CDH, in contrast, has been reported in a patient with a recessive FREM1mutation.The effect of the latter was supported by a mouse model.(72) Similarly, there have been somewhat conflicting reports on craniosynostosis in Wolf-Hirschorn syndrome, which includes deletions of 4p16.1. The effect of deletion of FGFRL1, located on 4p16.3 has been implicated in the development of craniosynostosis in both mouse models and humans.(73) In addition, a case report described a patient with a de novo 4p15 deletion, who presented with metopic synostosis.(74) However, the characteristic 4p16.1 was not included in the deletion and therefore this patient was considered to have a different phenotype.(74) In contrast, there have been several reports on patients with Wolf-Hirschorn who presented with CDH.(75-77)

**References**

1. Kaur R, Mishra P, Kumar S, Sankar MJ, Kabra M, Gupta N. Apert syndrome with congenital diaphragmatic hernia: another case report and review of the literature. Clin Dysmorphol. 2019;28(2):78-80.

2. Dap M, Bach-Segura P, Bertholdt C, Menzies D, Masutti JP, Klein O, et al. Variable phenotypic expression of Apert syndrome in monozygotic twins. Clin Case Rep. 2019;7(1):54-7.

3. Kosinski P, Luterek K, Wielgos M. Diaphragmatic hernia as an early ultrasound manifestation of Apert syndrome. Ginekol Pol. 2016;87(12):830.

4. Sobaih BH, AlAli AA. A third report of Apert syndrome in association with diaphragmatic hernia. Clin Dysmorphol. 2015;24(3):106-8.

5. Bulfamante G, Gana S, Avagliano L, Fabietti I, Gentilin B, Lalatta F. Congenital diaphragmatic hernia as prenatal presentation of Apert syndrome. Prenat Diagn. 2011;31(9):910-1.

6. Witters I, Devriendt K, Moerman P, van Hole C, Fryns JP. Diaphragmatic hernia as the first echographic sign in Apert syndrome. Prenatal Diagnosis: Published in Affiliation With the International Society for Prenatal Diagnosis. 2000;20(5):404-6.

7. Wallis-Crespo MC, Gilbert-Barness E. Pathology teach and tell: acrocephalosyndactyly type I (Apert syndrome). Fetal Pediatr Pathol. 2004;23(1):71-8.

8. Lajeunie E, Cameron R, El Ghouzzi V, de Parseval N, Journeau P, Gonzales M, et al. Clinical variability in patients with Apert's syndrome. J Neurosurg. 1999;90(3):443-7.

9. Martinez-Lage JF, Felipe-Murcia M, Navarro EG, Almagro MJ, Lopez-Guerrero AL, Perez-Espejo MA. Craniosynostosis in Kabuki syndrome. J Neurosurg Pediatr. 2010;6(2):198-201.

10. David G, Sillence D, Hardwick R, Opitz JM. A case of Kabuki (Niikawa-Kuroki) syndrome associated with manifestations resembling C-trigonocephaly syndrome. American journal of medical genetics Part A. 2004;130A(4):389-92.

11. Topa A, Samuelsson L, Lovmar L, Stenman G, Kolby L. On the significance of craniosynostosis in a case of Kabuki syndrome with a concomitant KMT2D mutation and 3.2 Mbp de novo 10q22.3q23.1 deletion. American journal of medical genetics Part A. 2017;173(8):2219-25.

12. Scott TM, Campbell IM, Hernandez-Garcia A, Lalani SR, Liu P, Shaw CA, et al. Clinical exome sequencing data reveal high diagnostic yields for congenital diaphragmatic hernia plus (CDH+) and new phenotypic expansions involving CDH. J Med Genet. 2021.

13. Silengo M, Lerone M, Seri M, Romeo G. Inheritance of Niikawa-Kuroki (Kabuki makeup) syndrome. Am J Med Genet. 1996;66(3):368.

14. Genevieve D, Amiel J, Viot G, Le Merrer M, Sanlaville D, Urtizberea A, et al. Atypical findings in Kabuki syndrome: report of 8 patients in a series of 20 and review of the literature. American journal of medical genetics Part A. 2004;129A(1):64-8.

15. van Haelst MM, Brooks AS, Hoogeboom J, Wessels MW, Tibboel D, de Jongste JC, et al. Unexpected life-threatening complications in Kabuki syndrome. Am J Med Genet. 2000;94(2):170-3.

16. Adam MP, Banka S, Bjornsson HT, Bodamer O, Chudley AE, Harris J, et al. Kabuki syndrome: international consensus diagnostic criteria. J Med Genet. 2019;56(2):89-95.

17. Mak CCY, Doherty D, Lin AE, Vegas N, Cho MT, Viot G, et al. MN1 C-terminal truncation syndrome is a novel neurodevelopmental and craniofacial disorder with partial rhombencephalosynapsis. Brain. 2020;143(1):55-68.

18. Miyake N, Takahashi H, Nakamura K, Isidor B, Hiraki Y, Koshimizu E, et al. Gain-of-Function MN1 Truncation Variants Cause a Recognizable Syndrome with Craniofacial and Brain Abnormalities. Am J Hum Genet. 2020;106(1):13-25.

19. McDonald-McGinn DM, Gripp KW, Kirschner RE, Maisenbacher MK, Hustead V, Schauer GM, et al. Craniosynostosis: another feature of the 22q11.2 deletion syndrome. American journal of medical genetics Part A. 2005;136A(4):358-62.

20. Unolt M, Kammoun M, Nowakowska B, Graham GE, Crowley TB, Hestand MS, et al. Pathogenic variants in CDC45 on the remaining allele in patients with a chromosome 22q11.2 deletion result in a novel autosomal recessive condition. Genet Med. 2020;22(2):326-35.

21. Unolt M, DiCairano L, Schlechtweg K, Barry J, Howell L, Kasperski S, et al. Congenital diaphragmatic hernia in 22q11.2 deletion syndrome. American journal of medical genetics Part A. 2017;173(1):135-42.

22. McDonald-McGinn DM, Tonnesen MK, Laufer-Cahana A, Finucane B, Driscoll DA, Emanuel BS, et al. Phenotype of the 22q11.2 deletion in individuals identified through an affected relative: cast a wide FISHing net! Genet Med. 2001;3(1):23-9.

23. Botto LD, May K, Fernhoff PM, Correa A, Coleman K, Rasmussen SA, et al. A population-based study of the 22q11.2 deletion: phenotype, incidence, and contribution to major birth defects in the population. Pediatrics. 2003;112(1 Pt 1):101-7.

24. Kruszka P, Li D, Harr MH, Wilson NR, Swarr D, McCormick EM, et al. Mutations in SPECC1L, encoding sperm antigen with calponin homology and coiled-coil domains 1-like, are found in some cases of autosomal dominant Opitz G/BBB syndrome. J Med Genet. 2015;52(2):104-10.

25. Robin NH, Feldman GJ, Aronson AL, Mitchell HF, Weksberg R, Leonard CO, et al. Opitz syndrome is genetically heterogeneous, with one locus on Xp22, and a second locus on 22q11.2. Nat Genet. 1995;11(4):459-61.

26. Bhoj EJ, Haye D, Toutain A, Bonneau D, Nielsen IK, Lund IB, et al. Phenotypic spectrum associated with SPECC1L pathogenic variants: new families and critical review of the nosology of Teebi, Opitz GBBB, and Baraitser-Winter syndromes. Eur J Med Genet. 2019;62(12):103588.

27. Wild KT, Gordon T, Bhoj EJ, Du H, Jhangiani SN, Posey JE, et al. Congenital diaphragmatic hernia as a prominent feature of a SPECC1L-related syndrome. American journal of medical genetics Part A. 2020;182(12):2919-25.

28. Bhoj EJ, Haye D, Toutain A, Bonneau D, Nielsen IK, Lund IB, et al. Phenotypic spectrum associated with SPECC1L pathogenic variants: new families and critical review of the nosology of Teebi, Opitz GBBB, and Baraitser-Winter syndromes. European Journal of Medical Genetics. 2019;62(12):103588.

29. Morris CA, Mervis CB, Paciorkowski AP, Abdul-Rahman O, Dugan SL, Rope AF, et al. 7q11.23 Duplication syndrome: Physical characteristics and natural history. American journal of medical genetics Part A. 2015;167A(12):2916-35.

30. Torniero C, Dalla Bernardina B, Novara F, Cerini R, Bonaglia C, Pramparo T, et al. Dysmorphic features, simplified gyral pattern and 7q11.23 duplication reciprocal to the Williams-Beuren deletion. Eur J Hum Genet. 2008;16(8):880-7.

31. Kriek M, White SJ, Szuhai K, Knijnenburg J, van Ommen GJ, den Dunnen JT, et al. Copy number variation in regions flanked (or unflanked) by duplicons among patients with developmental delay and/or congenital malformations; detection of reciprocal and partial Williams-Beuren duplications. Eur J Hum Genet. 2006;14(2):180-9.

32. Van der Aa N, Rooms L, Vandeweyer G, van den Ende J, Reyniers E, Fichera M, et al. Fourteen new cases contribute to the characterization of the 7q11.23 microduplication syndrome. Eur J Med Genet. 2009;52(2-3):94-100.

33. Berg JS, Brunetti-Pierri N, Peters SU, Kang SH, Fong CT, Salamone J, et al. Speech delay and autism spectrum behaviors are frequently associated with duplication of the 7q11.23 Williams-Beuren syndrome region. Genet Med. 2007;9(7):427-41.

34. Twigg SR, Kan R, Babbs C, Bochukova EG, Robertson SP, Wall SA, et al. Mutations of ephrin-B1 (EFNB1), a marker of tissue boundary formation, cause craniofrontonasal syndrome. Proc Natl Acad Sci U S A. 2004;101(23):8652-7.

35. van den Elzen ME, Twigg SR, Goos JA, Hoogeboom AJ, van den Ouweland AM, Wilkie AO, et al. Phenotypes of craniofrontonasal syndrome in patients with a pathogenic mutation in EFNB1. Eur J Hum Genet. 2014;22(8):995-1001.

36. Acosta-Fernandez E, Zenteno JC, Chacon-Camacho OF, Pena-Padilla C, Bobadilla-Morales L, Corona-Rivera A, et al. Extracranial midline defects in a patient with craniofrontonasal syndrome with a novel EFNB1 mutation. American journal of medical genetics Part A. 2020;182(5):1223-9.

37. Brooks AS, van Dooren M, Hoogeboom J, Gischler S, Willems PJ, Tibboel D. Congenital diaphragmatic hernia in a female patient with craniofrontonasal syndrome. Clin Dysmorphol. 2002;11(2):151-3.

38. Hogue J, Shankar S, Perry H, Patel R, Vargervik K, Slavotinek A. A novel EFNB1 mutation (c.712delG) in a family with craniofrontonasal syndrome and diaphragmatic hernia. American journal of medical genetics Part A. 2010;152A(10):2574-7.

39. McGaughran J, Rees M, Battin M. Craniofrontonasal syndrome and diaphragmatic hernia. Am J Med Genet. 2002;110(4):391-2.

40. Morris CA, Palumbos JC, Carey JC. Delineation of the male phenotype in carniofrontonasal syndrome. Am J Med Genet. 1987;27(3):623-31.

41. Vasudevan PC, Twigg SR, Mulliken JB, Cook JA, Quarrell OW, Wilkie AO. Expanding the phenotype of craniofrontonasal syndrome: two unrelated boys with EFNB1 mutations and congenital diaphragmatic hernia. Eur J Hum Genet. 2006;14(7):884-7.

42. Hurst J, Baraitser M. Craniofrontonasal dysplasia. J Med Genet. 1988;25(2):133-4.

43. Twigg SR, Matsumoto K, Kidd AM, Goriely A, Taylor IB, Fisher RB, et al. The origin of EFNB1 mutations in craniofrontonasal syndrome: frequent somatic mosaicism and explanation of the paucity of carrier males. Am J Hum Genet. 2006;78(6):999-1010.

44. Kawamoto HK, Heller JB, Heller MM, Urrego A, Gabbay JS, Wasson KL, et al. Craniofrontonasal dysplasia: a surgical treatment algorithm. Plastic and reconstructive surgery. 2007;120(7):1943-56.

45. Desai JJ, Nair SB, Pappachan S. Classic Cornelia de Lange syndrome with variant of unknown significance detected in NIPBL gene mutation: a case report. Egyptian Journal of Medical Human Genetics. 2021;22(1):1-6.

46. Xu Y, Sun S, Li N, Yu T, Wang X, Wang J, et al. Identification and analysis of the genetic causes in nine unrelated probands with syndromic craniosynostosis. Gene. 2018;641:144-50.

47. Gupta VS, Khan AM, Ebanks AH, Lally PA, Lally KP, Harting MT, et al. Cornelia de Lange syndrome and congenital diaphragmatic hernia. J Pediatr Surg. 2021;56(4):697-9.

48. Kline AD, Moss JF, Selicorni A, Bisgaard AM, Deardorff MA, Gillett PM, et al. Diagnosis and management of Cornelia de Lange syndrome: first international consensus statement. Nat Rev Genet. 2018;19(10):649-66.

49. Schirwani S, Novelli A, Digilio MC, Bourn D, Wilson V, Roberts C, et al. Duplications of GPC3 and GPC4 genes in symptomatic female carriers of Simpson-Golabi-Behmel syndrome type 1. Eur J Med Genet. 2019;62(4):243-7.

50. Li CC, McDonald SD. Increased nuchal translucency and other ultrasound findings in a case of simpson-golabi-behmel syndrome. Fetal Diagn Ther. 2009;25(2):211-5.

51. Villarreal DD, Villarreal H, Paez AM, Peppas D, Lynch J, Roeder E, et al. A patient with a unique frameshift mutation in GPC3, causing Simpson-Golabi-Behmel syndrome, presenting with craniosynostosis, penoscrotal hypospadias, and a large prostatic utricle. American journal of medical genetics Part A. 2013;161A(12):3121-5.

52. Chen E, Johnson JP, Cox VA, Golabi M. Simpson-Golabi-Behmel syndrome: congenital diaphragmatic hernia and radiologic findings in two patients and follow-up of a previously reported case. Am J Med Genet. 1993;46(5):574-8.

53. Neri G, Gurrieri F, Zanni G, Lin A. Clinical and molecular aspects of the Simpson-Golabi-Behmel syndrome. Am J Med Genet. 1998;79(4):279-83.

54. Piard J, Collet C, Arbez-Gindre F, Nirhy-Lanto A, Van Maldergem L. Coronal craniosynostosis and radial ray hypoplasia: a third report of Twist mutation in a 33 weeks fetus with diaphragmatic hernia. Eur J Med Genet. 2012;55(12):719-22.

55. Abulezz TA, Allam KA, Wan DC, Lee JC, Kawamoto HK. Saethre-Chotzen Syndrome: A Report of 7 Patients and Review of the Literature. Ann Plast Surg. 2020;85(3):251-5.

56. Alfi O, Donnell GN, Crandall BF, Derencsenyi A, Menon R. Deletion of the short arm of chromosome no.9 (46,9p-): a new deletion syndrome. Ann Genet. 1973;16(1):17-22.

57. Ng I, Chin W, Lim E, Tan E. An additional case of the recurrent 15q24.1 microdeletion syndrome and review of the literature. Twin Res Hum Genet. 2011;14(4):333-9.

58. Bettelheim D, Hengstschlager M, Drahonsky R, Eppel W, Bernaschek G. Two cases of prenatally diagnosed diaphragmatic hernia accompanied by the same undescribed chromosomal deletion (15q24 de novo). Clin Genet. 1998;53(4):319-20.

59. Van Esch H, Backx L, Pijkels E, Fryns JP. Congenital diaphragmatic hernia is part of the new 15q24 microdeletion syndrome. Eur J Med Genet. 2009;52(2-3):153-6.

60. Sharp AJ, Selzer RR, Veltman JA, Gimelli S, Gimelli G, Striano P, et al. Characterization of a recurrent 15q24 microdeletion syndrome. Hum Mol Genet. 2007;16(5):567-72.

61. El-Hattab AW, Smolarek TA, Walker ME, Schorry EK, Immken LL, Patel G, et al. Redefined genomic architecture in 15q24 directed by patient deletion/duplication breakpoint mapping. Hum Genet. 2009;126(4):589-602.

62. Liu Y, Mapow B. Coexistence of urogenital malformations in a female fetus with de novo 15q24 microdeletion and a literature review. Mol Genet Genomic Med. 2020;8(7):e1265.

63. Vasileiou G, Vergarajauregui S, Endele S, Popp B, Buttner C, Ekici AB, et al. Mutations in the BAF-Complex Subunit DPF2 Are Associated with Coffin-Siris Syndrome. Am J Hum Genet. 2018;102(3):468-79.

64. Knapp KM, Poke G, Jenkins D, Truter W, Bicknell LS. Expanding the phenotypic spectrum associated with DPF2: A new case report. American journal of medical genetics Part A. 2019;179(8):1637-41.

65. Das BB, Jayakumar KA, Young ML, Chan KC. Senning Procedure for Physiological Atrial Inversion With Left Atrial Isomerism. JACC Case Rep. 2019;1(4):516-22.

66. Loeys BL, Chen J, Neptune ER, Judge DP, Podowski M, Holm T, et al. A syndrome of altered cardiovascular, craniofacial, neurocognitive and skeletal development caused by mutations in TGFBR1 or TGFBR2. Nat Genet. 2005;37(3):275-81.

67. Lobaton GO, Chen YJ, Jelin E, Garcia AV. Unusual case of delayed congenital diaphragmatic hernia in Loeys-Dietz syndrome: a case report. J Surg Case Rep. 2021;2021(1):rjaa604.

68. Srour M, Caron V, Pearson T, Nielsen SB, Levesque S, Delrue MA, et al. Gain-of-Function Mutations in RARB Cause Intellectual Disability with Progressive Motor Impairment. Hum Mutat. 2016;37(8):786-93.

69. Srour M, Chitayat D, Caron V, Chassaing N, Bitoun P, Patry L, et al. Recessive and dominant mutations in retinoic acid receptor beta in cases with microphthalmia and diaphragmatic hernia. Am J Hum Genet. 2013;93(4):765-72.

70. Vissers LE, Cox TC, Maga AM, Short KM, Wiradjaja F, Janssen IM, et al. Heterozygous mutations of FREM1 are associated with an increased risk of isolated metopic craniosynostosis in humans and mice. PLoS Genet. 2011;7(9):e1002278.

71. Dawson AJ, Hovanes K, Liu J, Marles S, Greenberg C, Mhanni A, et al. Heterozygous intragenic deletions of FREM1 are not associated with trigonocephaly. Clin Dysmorphol. 2021;30(2):83-8.

72. Beck TF, Veenma D, Shchelochkov OA, Yu Z, Kim BJ, Zaveri HP, et al. Deficiency of FRAS1-related extracellular matrix 1 (FREM1) causes congenital diaphragmatic hernia in humans and mice. Hum Mol Genet. 2020;29(6):1054.

73. Rieckmann T, Zhuang L, Fluck CE, Trueb B. Characterization of the first FGFRL1 mutation identified in a craniosynostosis patient. Biochim Biophys Acta. 2009;1792(2):112-21.

74. Alesi V, Barrano G, Morara S, Darelli D, Petrilli K, Capalbo A, et al. A previously undescribed de novo 4p15 deletion in a patient with apparently isolated metopic craniosynostosis. American journal of medical genetics Part A. 2011;155A(10):2543-51.

75. van Dooren MF, Brooks AS, Hoogeboom AJ, van den Hoonaard TL, de Klein JE, Wouters CH, et al. Early diagnosis of Wolf-Hirschhorn syndrome triggered by a life-threatening event: congenital diaphragmatic hernia. American journal of medical genetics Part A. 2004;127A(2):194-6.

76. Casaccia G, Mobili L, Braguglia A, Santoro F, Bagolan P. Distal 4p microdeletion in a case of Wolf-Hirschhorn syndrome with congenital diaphragmatic hernia. Birth Defects Res A Clin Mol Teratol. 2006;76(3):210-3.

77. Basgul A, Kavak ZN, Akman I, Basgul A, Gokaslan H, Elcioglu N. Prenatal diagnosis of Wolf-Hirschhorn syndrome (4p-) in association with congenital diaphragmatic hernia, cystic hygroma and IUGR. Clin Exp Obstet Gynecol. 2006;33(2):105-6.
